# Oncolytic Virotherapy with Myxoma Virus

**DOI:** 10.3390/jcm9010171

**Published:** 2020-01-08

**Authors:** Masmudur M. Rahman, Grant McFadden

**Affiliations:** Center for Immunotherapy, Vaccines and Virotherapy, Biodesign Institute, Arizona State University, Tempe, AZ 85287, USA

**Keywords:** Myxoma virus, Oncolytic Virus, Oncolytic, Virotherapy, cancer treatment

## Abstract

Oncolytic viruses are one of the most promising novel therapeutics for malignant cancers. They selectively infect and kill cancer cells while sparing the normal counterparts, expose cancer- specific antigens and activate the host immune system against both viral and tumor determinants. Oncolytic viruses can be used as monotherapy or combined with existing cancer therapies to become more potent. Among the many types of oncolytic viruses that have been developed thus far, members of poxviruses are the most promising candidates against diverse cancer types. This review summarizes recent advances that are made with oncolytic myxoma virus (MYXV), a member of the Leporipoxvirus genus. Unlike other oncolytic viruses, MYXV infects only rabbits in nature and causes no harm to humans or any other non-leporid animals. However, MYXV can selectively infect and kill cancer cells originating from human, mouse and other host species. This selective cancer tropism and safety profile have led to the testing of MYXV in various types of preclinical cancer models. The next stage will be successful GMP manufacturing and clinical trials that will bring MYXV from bench to bedside for the treatment of currently intractable malignancies.

## 1. Introduction

Cancer is the leading cause of death globally, with an estimated mortality of 9.6 million in 2018 (World Health Organization). Conventional cancer treatment regimens typically include one or more modality such as surgery, radiotherapy and chemotherapy. However, with these treatments it becomes progressively harder to cure or prolong patient life if the cancer is not diagnosed at a sufficiently early stage, has metastasized at secondary sites, and/or has relapsed into therapy-resistance after initial treatment. Currently available new therapies include immunotherapy, targeted therapy, hormone therapy, stem cell therapy, engineered lymphocytes and, finally, virotherapy with oncolytic viruses (OVs). Among these, OVs have emerged as an exceedingly promising modality for cancer treatment based on their multi-mechanistic advantages against diverse types of cancer: the selective infection and killing of cancer cells, the ability in some cases to target cancer at metastatic sites, the release of tumor-associated antigens, triggering of novel anti-tumor innate and adaptive immune responses, and the activation and recruitment of immune cells into the tumor microenvironment (TME). Furthermore, genetic modification can further enhance the antitumor activity of many OVs and thus increase the suitability for combination with conventional or newer therapies. OVs that are in various stages of preclinical or clinical development are members of both RNA and DNA viruses [1,2,3,4,5,6]. After successful testing in pre-clinical models, many OVs are now in varying stages of clinical trials with human cancer patients [7]. The first genetically modified OV, an engineered adenovirus named H101, was approved in China in 2005 for the treatment of head and neck cancer [8]. Recently, HSV-1 based Talimogene Laherparepvec (T-VEC) OV was approved in 2015 in the USA and Europe for the treatment of non-resectable metastatic melanoma [9].

Myxoma virus (MYXV) is the prototypic member of the Leporipoxvirus genus of the *Poxviridae* family of DNA viruses [10,11]. Like all poxviruses, the dsDNA genome of MYXV is relatively large (161.8 kbp) and encodes about 171 viral genes [12]. About one-third of these genes, located mostly in the central region of the genome, are highly conserved in all poxviruses and are required for housekeeping functions like viral replication, transcription, translation and virion assembly. The remaining two-thirds of the viral genes, that tend to be clustered at or near the terminal genomic regions, are unique to specific poxviruses and are involved in subverting the host immune responses and other anti-viral pathways [13,14]. These viral genes are also known as host immune modulators that are dedicated to pathogenesis and virus propagation within an immunocompetent host, and often dictate whether a given poxvirus can infect multiple species as they target related host determinants from diverse species. For example, vaccinia virus (VACV), a prototypic member of orthopoxvirus, can infect multiple host species, whereas MYXV and Ectromelia are highly restricted to rabbits and mice, respectively [15]. The natural hosts of MYXV are lagomorphs of the Sylvilagus genus, such as the Brazilian tapeti and American brush rabbits, where the virus has co-evolved as a relatively nonpathogenic infection, likely for millions of years. In these host species, MYXV only causes benign cutaneous lesions and spreads from host-to-host by biting arthropod vectors, without any association with overt disease [16,17]. In stark contrast to Sylvilagus rabbits, however, MYXV causes a lethal disease called myxomatosis in the *Oryctolagus cuniculus* (European rabbit) species. This extreme pathogenicity of MYXV in European rabbits was first documented in the late 19th century when the virus was first transmitted by mosquito vectors from wild Sylvilagus rabbits (i.e., the natural host) to captive Oryctolagus rabbits that had been imported to South America [18]. The molecular and genetic basis of this extreme pathogenicity for MYXV in a single host, European rabbits, and absence of pathogenicity in the natural evolutionary tapeti/brush rabbits, is still not well understood. Due to its extreme lethality against European rabbits, MYXV was released in the 1950s in Australia and Europe to control the feral European rabbit populations, that had bred to environmentally damaging levels in the wild. During this deliberate release of MYXV in Australia, it was demonstrated that the virus is nonpathogenic for all nonrabbit animals tested, including humans [19,20]. The topic of how MYXV is being developed as an OV to treat human cancer has been summarized previously [21], and this review will focus on developments since then (Table 1).

## 2. MYXV Tropism for Cancer Cells 

Unlike many other types of viruses, poxviruses like MYXV, in general, do not rely on a specific cell surface receptor for binding, which allows them to attach to, and at least initiate infection of, diverse cell types originating from different species [32]. However, there are few exceptions; for example, both MYXV and VACV are unable to bind and infect primary CD34^+^ hematopoietic stem cells [33,34]. Poxviruses enter cells by a multistep process consisting of cell attachment, hemi-fusion of the viral envelop with the cell surface membrane or internalized endosomal structures, and then virion core injection into the cytoplasm. Most of these initial infection processes are documented from studies on VACV [35,36,37,38]. The first step is relatively ubiquitous virion binding to the mammalian cell surface, which is mediated by at least four virus-encoded attachment proteins: D8, A27, H3 and A26 [35]. D8 binds chondroitin, A27 and H3 bind heparan, and A26 binds laminin [39,40,41,42]. This poxvirus binding to cells also involves interaction with integrin β1 and CD98 receptor molecules, and further activation of several serine/threonine kinases that are involved in the process of micropinocytosis or fluid phase endocytosis [43,44]. The next step of virus entry is internalization into the cell cytoplasm after membrane fusion. The virion entry fusion complex (EFC) of VACV that mediates the membrane fusion events consists of a complex of at least 11 viral proteins [36]. The fusion of the mature virion (MV) membrane with the plasma and/or endosomal membranes results in the deposition of the viral core into the cytoplasm of infected cells. Fusion is activated by low pH, but no specific cell receptor that activates fusion has yet been identified, which is why the binding/entry event for most poxviruses is so promiscuous against multiple classes and species of mammalian cells. 

Nevertheless, comparing the infectivity of VACV and MYXV in different human cancer cell lines has suggested that some differences do exist in terms of how these two poxviruses interact with cancer cells and primary human leukocytes. For example, VACV demonstrated lower binding to human multiple myeloma cell lines U266 and HuNS1 compared to MYXV [45]. For either VACV or MYXV, binding to different cancer cell types varies significantly, mainly because of the changes in the binding determinants on the surface of target cells. For example, heparin sulphate is not a major determinant for the binding of MYXV or VACV to nonadherent HuNS1 myeloma cells but is required for binding to another human myeloma cell line, U266, and many transformed adherent cell lines [45,46]. Another example of a host cell tropism determinant is the extracellular matrix protein laminin, which is not required for MYXV binding to tested HeLa and myeloma cell lines but is required for VACV to bind HeLa cells and many transformed human or murine cell lines tested in culture [40,45]. Both MYXV and VACV demonstrated similar levels of binding to primary human leukocytes derived from blood or bone marrow samples [45]. However, virion binding to these primary leukocyte populations was not blocked by either heparin or laminin, suggesting that binding to these cells is independent of heparin sulphate or laminin [34,45]. In contrast to human CD34^+^ stem cells, MYXV was able to bind naïve nonactivated primary human T lymphocytes but aborted at this early step without causing subsequent infection. This is a rare example of poxvirus binding to a target cell without entering and inducing early virus gene expression. However, following the activation of these primary T cells, for example with anti-CD3/CD28 or nonspecific T cell activators, the bound MYXV was able to now enter and initiate virus gene expression, allowing it to proceed to a complete virus replication cycle [47]. These results suggest that MYXV binding and infection of primary lymphocytes may vary dramatically with cell type and their activation status.

## 3. Role of Intracellular Signaling in Cancer Cell Tropism

As a way of protecting themselves against host immune surveillance, cancer cells have been selected to circumvent or disrupt many innate immune sensing pathways, including anti-viral signaling. These genetic defects in cancer cells collectively represent an Achilles heel that allows them to be preferentially infected by many classes of viruses, including OVs. However, their non-transformed cellular counterparts, that maintain fully functional antiviral sensing/signaling, are frequently more resistant to infection by the same virus. OVs exploit these inherent defects found in cancer cells to mediate selective infection and replication, but the actual level of productive infection varies dramatically among viruses and cancer cell types. Particularly for poxviruses, this increased tropism for cancer cells depends on the ability of virus-encoded proteins to modulate the antiviral signaling pathways still operational in cancer cells. Large DNA poxviruses, such as VACV and MYXV, encode dozens of host modulatory proteins that make them highly effective in terms of modulating multiple cellular pathways after entering the cells [14]. 

MYXV, a virus that has largely adapted to rabbits as a host, can nevertheless infect different types of cancer cells originating from diverse species [21]. The intracellular signaling pathways that regulate MYXV replication in human cancer cells identified so far include: Protein kinase B (also known as Akt), Protein kinase R (PKR), Sterile alpha motif domain containing 9 (SAMD9), and various host DEAD-box RNA helicases. Due to the cellular transformation, Akt is often constitutively phosphorylated at different levels in many cancer cells types. MYXV preferentially replicates in cancer cell lines where both Thr308 and Ser473 are phosphorylated [48]. However, when only Thr308 is phosphorylated, the MYXV-encoded ankyrin repeat containing protein M-T5 is exploited for the subsequent phosphorylation of Ser473 and release of the block against virus replication [49]. For cancer cell lines where Akt is not phosphorylated at either of these sites, MYXV is frequently not able to replicate, and they are classified as non-permissive cancer cell lines [48]. Thus, in the absence of M-T5 protein, MYXV is not able to replicate in those human cancer cell lines with hemi-phosphorylated Akt [50]. Another cellular protein associated with the regulation of MYXV replication in human cancer cells is SAMD9, an interferon regulated protein, targeted by MYXV protein M062, a functional homolog of the C7L family of host range proteins from orthopoxviruses [51]. Following knockout of the *M062* gene, MYXV is unable to produce significant levels of the progeny virus in diverse human cancer cell lines [51]. SAMD9 and SAMD9L, a paralog of SAMD9 that is encoded by some mammals, is also inhibited by other members of the C7L family of proteins, such as K1, C7 and CP77 [52,53,54,55]. PKR is another important and rapidly evolving host cell antiviral kinase in almost every cell type, including cancer cells. Thus, inhibition of PKR is key for MYXV replication in both rabbit and non-rabbit cell lines. MYXV encoded dsRNA binding protein M029, an ortholog of VACV E3 family of proteins, is essential for MYXV replication in all human cells tested [56]. In a recent study, it was demonstrated that members of the DEAD-box containing RNA helicase superfamily of proteins also play critical regulatory roles for MYXV tropism in human cancer cells [57]. In addition to these known cellular factors, cancer cells’ inherent inability to induce a synergistic antiviral state against type I IFN plus TNF, and their mutational inactivation of genes like *RB* and *p53* also allow MYXV infection and replication in diverse types of cancer cells [58,59,60]. It is likely that many more antiviral pathways that regulate MYXV tropism in human cancer cells are still to be discovered.

## 4. MYXV Oncolytic Killing of Cancer Cells

Most viruses have acquired diverse mechanisms to evade host-regulated cell death, such as apoptosis, necroptosis and pyroptosis, for example, by encoding proteins that target these pathways [61]. Although eventually the virus-infected cells die and are cleared by the immune system, the battle between virus and host cells can delay the kinetics and alter the type of cell death. Like many poxviruses, MYXV also encodes proteins that prevent or delay the induction of virus-induced cell death. In some studies, it was shown that deletion of these viral death modulator genes can enhance cancer cell killing, and thus can be a more effective oncolytic virus platform. The expectation is that modified OVs that operate through the enhanced cell killing of cancer cells can more robustly expose tumor antigens and induce stronger anti-tumor immune responses [62]. For example, MYXV infection of human multiple myeloma (MM) cell lines and primary patient-derived MM cells results in unusually rapid cell death. This killing was independent of viral replication, but rather appeared to occur through virus-mediated induction of programmed cell death, which leads to the ligand-independent activation of caspase-8 [63,64]. In MYXV, the viral proteins that have been identified so far as regulators of cell death include: M-T5, M13, M11, and Serp2. M11L is a key anti-apoptotic protein member of the Bcl2 superfamily encoded by MYXV and acts as a potent inhibitor of intrinsic apoptosis [65]. M11L interacts with several host pro-apoptotic Bcl-2 proteins, including Bak, Bax, Bim and Bid [66,67]. Structural studies revealed that M11L adopts a monomeric Bcl2 fold to interact with pro-apoptotic Bcl-2 proteins Bak and Bax [66,68]. As discussed later, the MYXV M11L-knockout construct showed enhanced oncolytic activity against glioma in vitro and in vivo [24]. Apart from M11L, the involvement of other viral proteins in modifying cell death pathways was confirmed by studies using the recombinant viruses not expressing the selected proteins. For example, the MYXV M-T5-knockout virus infection of human cancer cell lines resulted in the selective activation of caspase 3 [50]. Similar activation of host cell caspases was also observed after infection with MYXV deleted for the *M013* gene that expresses a PYRIN domain-containing protein [69]. The MYXV genome also encodes serine protease inhibitors (serpins) that antagonize the function of various host caspases. Among these viral serpins, SERP1 has been shown to inhibit caspase 1 and granzyme B [70]. Another MYXV encoded serpin that regulates caspase activity is SERP2. SERP2 has been characterized as a weak inhibitor of granzyme B and interleukin-1beta-converting enzyme (ICE, caspase-1), in contrast to CrmA, encoded by CPXV [71]. Deletion of *Serp2* gene from the MYXV genome results in a virus that selectively induces apoptosis in rabbit lymphocytes. This was also observed in canine tumor cells, where Serp2 KO MYXV induced more cytopathic effects and evidenced increased oncolytic activity [72].

## 5. Oncolytic Virotherapy for Solid Tumors

### 5.1. Small Cell Lung Cancer 

Lung cancer is the second most common cancer in both men and women. Among the various forms of lung cancers, the two main types are: non-small-cell lung cancer (NSCLC) and small-cell lung cancer (SCLC). In general, about 10–15% of all lung cancers are SCLC. SCLC is considered a very aggressive and lethal malignancy due to its early metastatic dissemination [73]. With SCLC, unfortunately most of the patients are diagnosed late with metastatic disease, and with current treatment options the general 5 year survival rate is only 6%. The SCLC-associated invasive tumor growth and early metastatic dissemination are mainly driven by the mutational inactivation of RB1 and p53. Patients with limited stage (LS) or extensive stage (ES) SCLC are commonly given platinum-based chemotherapy, such as cisplatin and carboplatin, with the non-platinum agent etoposide as first-line treatment [74]. Immunotherapy using an anti-PD1 antibody, prolonged survival compared to standard chemotherapy against NSCLC; however, for SCLC the response rate to immunotherapy remains low, in the range of 15–30% [75]. Various OVs have been tested as potential viral immunotherapies against NSCLC, including VSV, measles virus (MV), VACV and adenovirus [76]. However, only a small number of OVs are in human clinical trials against lung cancer indications. One of the obstacles for OVs is the delivery of sufficient therapeutic virus to the SCLC tumors and metastatic sites. 

In vitro, MYXV can productively infect and replicate in both adherent monolayer and nonadherent (i.e., floating spheroid) human SCLC cell lines, whereas normal human lung epithelial cells were essentially nonpermissive to MYXV [22]. In the tested human SCLC cell lines, MYXV infection caused cell death and increased the early release of ATP, a marker for induced immunogenic cell death (ICD). In addition, like human SCLC cell lines, MYXV also productively infected patient-derived primary SCLC cells collected by bronchoscopy [22]. The oncolytic activity of MYXV against SCLC was then tested in an immunocompetent genetically engineered mouse model (GEMM). In this model, the conditional knockouts of *p53*, *Rb* and *P130* using intratracheal delivery of adenovirus Cre-recombinase (Ad-Cre) led to the development of multiple SCLC tumor foci in the lungs [77]. These murine tumors represented features characteristic of end-stage human lung SCLC. When tested for immune cell infiltration, the lung tumor lesions exhibited minimal CD45^+^ and CD3^+^ immune cell populations, which also is reminiscent of the situation with primary human SCLC tumor biopsies [22]. Using this murine SCLC mouse model, syngeneic murine SCLC cell lines that were either adherent or floating spheroid phenotypes were generated and infected with MYXV to test for oncolytic killing. Like human SCLC cell lines, these murine SCLC cell lines were also productively infected with MYXV and showed an enhanced early release of ATP after MYXV infection [22]. Furthermore, a cisplatin-resistant murine SCLC cell line, which was generated by continuous exposure to cisplatin, was also infected by MYXV, suggesting that even drug-relapsed SCLC patients might be treatable with oncolytic MYXV. To test whether MYXV can infect SCLC cells in vivo within the tumor bed in situ in GEMM SCLC mice, the virus was delivered into the lung by intranasal instillation three months after tumor induction via intratracheal Ad-Cre delivery [22]. In the MYXV-treated lungs, MYXV replication was detected three days post-delivery using the expression of FLuc as a reporter, however, the signal was mostly eliminated by seven days post-delivery. More importantly, using this delivery method, MYXV replication was not detected in any other organs, again supporting the safety profile of MYXV. When the tumors were collected after 30 days of viral delivery and stained with CD45^+^ positive leukocytes, significant increases in immune cell infiltration were observed in the tumor bed [22]. These results suggest that MYXV enhanced the immune-stimulatory responses in vivo in the SCLC tumor bed. Based on these findings, a survival study was performed in this model by intranasal delivery of MYXV as therapy, with or without cisplatin. In this test, MYXV alone, or in combination with cisplatin, significantly enhanced the survival of mice compared to control PBS or treatment with cisplatin alone [22]. These results demonstrate the potential of using MYXV as a virotherapy and immunostimulatory treatment agent for SCLC.

### 5.2. Ovarian Cancer

Ovarian cancer (OC) is the sixth most prevalent cancer in women, with a high relapse rate. The five year relative survival rate for all types and stages of ovarian cancer is 47%, making it the most lethal of the gynecologic malignancies (American Cancer Society). OVs, in combination with immunotherapeutic agents such as Immune Checkpoint Inhibitors (ICIs), has shown promise in preclinical models and clinical trials. In a recent study, it was shown that, in select ovarian cancer cell lines, STING-dependent innate immune signaling pathways were defective, allowing cancer cells to become more susceptible to OVs. Surprisingly, the dsRNA activated RIG-I/MDA5 innate immune cytokine production pathway was functional in these cells [78]. Among the OVs, Measles, Adenovirus, VACV and Reovirus have been used in clinical trials [79]. MYXV has been tested in both human EOC cell lines and primary human EOC cells, isolated directly from patient ascites, which are cultured as suspension EOCs or spheroids [80]. In a later study, it was shown that although MYXV infected and reduced the viability of adherent monolayer cultured cells, in 3D spheroids the virus replication was restricted [81]. This is possibly because, in spheroids, Akt signaling becomes downregulated, which directly affected MYXV replication and oncolytic efficacy [80]. However, when the spheroids were reattached, the activated AKT level increased and also enhanced MYXV infection and oncolytic killing of cells. This was also observed with patient ascites derived EOC cells grown in spheroids, which showed MYXV entry but no cytopathic effects. The direct infection of freshly-collected ascites demonstrated that more than 50% patient samples were sensitive to MYXV-mediated oncolytic cell killing [81]. In a recent study, the combination of MYXV oncolytic virotherapy with chemotherapy cisplatin was tested in a disseminated OC mouse model. In this model, delivery of MYXV first, and subsequent treatments with cisplatin increased, the survival of mice. Another interesting observation was that OC patient ascites-associated CD14^+^ myeloid cells, when infected in vitro with MYXV, reduced the secretion of IL-10, a signature of the immunosuppressive tumor environment [23].

### 5.3. Glioblastoma

Glioblastoma, also known as glioblastoma multiforme (GBM), is one of the most aggressive types of cancers that starts in astrocytes that support nerve cells. The treatments for GBMs are surgery, followed by radiation and chemotherapy. But these treatments are largely ineffective and the median survival time for GBM is only 15 to 16 months [82]. Among the chemotherapy options, temozolomide (TMZ) improves the survival of patients with GBM, however, the development of resistance to GBM is very common and, thus, newer therapies are urgently required. Another barrier to GBM therapy is the intra-tumoral heterogeneity in the tumor-initiating cell population, which quickly develop a resistance to conventional therapies. OVs, in this case, are ideal candidates, as they can overcome the genetic complexity of GBM cell populations. A variety of OVs have been tested against GBM, and several clinical trials are currently ongoing to test them in patients [83]. MYXV has shown potent oncolytic activity in orthotopic preclinical glioma models in which human glioma xenografts were implanted [84]. MYXV was also able to efficiently infect and kill primary human gliomas obtained from surgical specimens cultured ex vivo [84]. In an immunocompetent rat model with Racine gliomas, MYXV, in combination with rapamycin, prolonged survival compared to MYXV alone. This enhanced effect of rapamycin was likely due to reduced type I IFN responses and the infiltration of NK cells and macrophages into MYXV-treated gliomas, suggesting that immunocompetent host modulation of the immune system is required for effective MYXV oncolytic virotherapy [85].

MYXV oncolytic virotherapy was tested against brain tumor-initiating cells (BTICs) for GBM [86]. BTICs were isolated from human gliomas that were cultured under neurosphere conditions and retained stem-cell-like properties. When both TMZ-resistant and -sensitive BTICs were tested, MYXV was able to infect and kill both types of BTIC lines [86]. MYXV infection and replication was further enhanced when BTIC cells were pretreated with rapamycin. The BTIC cell lines were used in a xenograft SCID mouse model. In this model, intratumoral delivery of MYXV, in combination with rapamycin, only provided long-term survival for some of the BTIC lines. This was correlated with the observations that, in the sensitive cells, MYXV infection decreased the expression of stem cell markers both in vitro and in vivo [86]. In contrast, MYXV showed modest activity against patient-derived, brain tumor-initiating cells (BTICs) [87]. In order to improve the oncolytic activity of MYXV against BTICs, a combination of chemotherapeutics with MYXV were tested. In this assay, a library of 73 compounds that are in clinical use or preclinical development was screened, and led to the identification of additional compounds that worked synergistically with MYXV in vitro [87]. Among the different compounds that were tested, axitinib, rofecaxib and pemetrexed showed the highest effect against BTICs in combination with MYXV [87]. 

To further study the impact of MYXV virotherapy against malignant glioma, mouse glioma cell lines derived from C57BL/6J NPcis mice (*Trp53*^+/−^/*Nfl*^+/−^), which spontaneously developed high-grade gliomas and recapitulated many clinical phenotypes of the human disease, were tested in syngeneic C57BL/6J mice. These mouse glioma cell lines were susceptible to in vitro MYXV infection, replication and killing [88]. However, intracranial injection of MYXV in the orthotopically grafted mouse gliomas failed to result in viral replication or treatment efficacy, as virus replication was cleared within seven days post-delivery [88]. In order to find out whether type I IFN signaling is involved in the reduction in MYXV replication and activity, an IRF9 knockdown cell line that did not respond to IFN was tested in this model. However, even in this cell-line-derived glioma, the in vivo MYXV virotherapy was not improved, suggesting that an antiviral state independent of glioma IFNα/β signaling might function against MYXV [88]. To further enhance the oncolytic activity of MYXV, a modified MYXV strain lacking the anti-apoptotic M011L (vMyx-M011LKO) was tested against human and mouse BTICs. In both human and mouse BTICs, infection with vMyx-M011LKO virus significantly enhanced the killing of cells by the activation of caspase 3/7 [24]. Human BTICs were tested in xenograft NSG mouse (NOD scid gamma) lacking mature B and T cells. Interestingly, unlike xenograft human BTICs in SCID mice, in NSG mice both wild-type and M11LKO MYXV did not show any survival benefits [24]. This is possibly because SCID mice are not as immunocompromised as NSG mice. In order to test the oncolytic activity of M11LKO MYXV in an immunocompetent mouse model, murine BTIC line mBTIC0309 was engrafted in immunocompetent C57BL/6J mice. In this model, the treatment of murine BTICs with M11LKO MYXV prolonged survival of mice compared to identical cohorts with wild-type MYXV [24]. Again, using the same murine BTIC line in NSG mice, the survival benefit with this MYXV construct was lost, suggesting the critical importance of immune responses against the viral infection, in order to induce clearance of the tumor. Further, in this immunocompetent BTIC model, when TMZ was combined with M11LKO virus, there was further prolonged survival of mice with only a single dose of virus and a subsequent five doses of TMZ [24]. Again, this survival benefit was not observed in NSG mice. In immunocompetent mice, robust activation of caspase 3 and Ki-67 was observed in the brain sections collected from mice treated with a combination of M11LKO and TMZ. However, this activation of caspase was not observed with wild-type or control UV-inactivated virus, suggesting the importance of caspase activation for BTIC tumor clearance in an immunocompetent host.

### 5.4. Gallbladder Cancer 

Gallbladder cancer (GBC) is rare but the most common biliary tract malignancy. The chances of curing gallbladder cancer are high if it is diagnosed at its earliest stage, but most gallbladder cancers are diagnosed late, after it spreads, due to a lack of early signs and symptoms. Surgery, radiation and chemotherapy are the most common types of treatment for GBC. However, metastatic gallbladder cancer is difficult to cure and the median survival for advanced stage cancer is less than a year [89]. Not many OVs have been tested against GBC. MYXV oncolytic activity has been assessed against human GBC cell lines in vitro and in vivo. MYXV infection of all the tested GBC cell lines allowed viral replication and cell killing, which was further enhanced with the treatment of rapamycin [25]. The level of activated AKT is one of the determinants of MYXV tropism in these cell lines. In the CD-1 nude mice xenograft model, either MYXV alone or in combination with rapamycin did not show efficacy when compared to a xenograft of human glioma cell line [25]. It was found that, in this model, a higher expression of collagen IV in the GBC tumors physically blocked MYXV intratumoral distribution. Subsequently, it was found that hyaluronan enhanced MMP-9 expression and AKT activation, which eventually also increased the oncolytic activity of MYXV for GBC in vitro and provided a survival benefit for xenograft mice in vivo [25]. In another study, it was shown that, in the same xenograft tumor model, the systemic delivery of MYXV using bone-marrow-derived stem cells (BMSCs) enhanced the oncolytic activity of MYXV [90]. However, further studies will require an improvement in the clearance of the tumor and a long-term survival benefit. The development of the syngeneic GBC mouse model will further allow for testing the efficacy of oncolytic virotherapy. 

### 5.5. Melanoma 

Melanoma is the leading cause of death from skin disease. Failure to diagnose early may lead to the development of metastatic melanoma that spreads from the skin to other parts of the body, such as the lymph nodes, liver, lungs, bones and brain. Metastatic melanoma is much harder to treat and the average five year survival rate for stage 4 metastatic melanoma is only 15–20% [91]. Apart from surgery, radiation, immunotherapy or chemotherapy are used to treat metastatic melanoma. Various OVs have been tested against melanoma [92]. Currently, T-VEC is the only OV approved in the USA and Europe for the treatment of advanced melanoma. T-VEC is a recombinant HSV (type 1) that has been engineered to contain mutations in the viral proteins ICP34.5 and ICP 47, and also to express human GM-CSF [9]. Other OVs tested against melanoma are HSV-1 mutant HF-10, coxsackieviruses (CVA21), Reovirus, VACV and Adenovirus, which all have all been tested in patients under different phases of clinical trial. MYXV was assessed against melanoma using B16F10 mouse melanoma cells in a syngeneic immunocompetent mouse model [93]. MYXV, alone or combination with rapamycin treatment ex vivo, inhibited the development of lung metastasis [93]. MYXV was also tested in a metastatic melanoma brain tumor syngeneic mouse model. In this model, B16.SIY melanoma cells were infused intracranially in C57BL/6 mice. However, multiple injections of MYXV in combination with rapamycin and/or concurrent T-cell immunotherapy were required to improve the overall outcome [26]. This was based on the finding that the presence of cytotoxic lymphocytes in the tumor bed can improve cancer patients’ survival and also decrease metastasis [26,94]. 

OVs can provide a unique platform to express immune-stimulating cytokines directly in the tumor bed. A recombinant MYXV-expressing murine, IL-15 (vMyx-IL-15), was tested in an immunocompetent melanoma model [27]. Intratumoral injection of this IL-15-armed MYXV in a subQ B16F10 melanoma model prolonged the survival of mice compared to the control, unarmed MYXV. Histological analysis of the tumor identified more inflammation in the tumor bed due to the infiltration of neutrophils. Since the co-expression of IL-15 with the α subunit of IL-15 receptor enhances the stability and bioactivity of IL-15, a new recombinant MYXV was constructed, expressing secreted IL-15Rα-IL-15 fusion protein. When this recombinant MYXV was tested in an immunocompetent B16F10 subQ model in wild-type or RAG^-/-^ background mice, tumor growth was attenuated and prolonged the survival of mice compared to the vMyx-IL-15 or unarmed MYXV [28]. Histological analysis of tumors in C57BL/6 mice showed strong a infiltration of both NK cells and CD8+ T cells in response to vMyx-IL-15Rα-IL-15 virus infection [28].

## 6. Oncolytic Virotherapy for Hematologic Malignancies 

Hematologic malignancies are cancers that begin in the cells of blood-forming tissues, such as bone marrow or different leukocytes of the immune system. Some examples of hematologic cancers are: multiple myeloma (MM), acute myeloid leukemia (AML), lymphomas and myelodysplastic syndromes. New cases of hematologic malignancies are expected to account for 10% of new cancer cases diagnosed in the USA in 2019 (Cancer facts and figures, 2019. American Cancer Society, 2019). Despite the overall increase in survival rates, more than 9% of cancer-related deaths are from these diseases. This is mainly because of the relapse of the disease after treatment with conventional therapies, including autologous hematopoietic stem cell transplant (HSCT). Numerous reports show that transplanted autografts frequently still contain cells that constitute minimal residual disease (MRD). Various purging strategies have been tested in the past to reduce MRD in the transplant sample, and sometimes to enrich the CD34^+^ stem cells [95,96]. However, existing methods do not completely eliminate all contaminating cancer cells from the autografts. The finding that MYXV is safe for normal hematopoietic stem cells because of virions’ inability to bind to CD34^+^ stem cells led to the testing of MYXV against AML and MM in preclinical cancer models. In these studies, the treatment of human AML or MM cells ex vivo with MYXV prior to xenotransplantation into NSG mice prevented the subsequent engraftment of cancer cells in the recipient host, suggesting that MYXV can be used to selectively delete various classes of cancer cells from autologous HSCT samples [64,97]. OVs have also been used to directly target cancer cells in their niches. For example, the measles virus is administered systemically via intravenous route in MM patients [98]. In a syngeneic mouse model of MM, the systemic delivery of MYXV significantly reduced tumor burden and prolonged the survival of mice [30]. The ability of MYXV to target MM niches was also tested using ex vivo virotherapy with bone marrow (BM) leukocytes in immunocompetent mice, using an allogeneic mouse–mouse transplant model [29]. In this case, the mouse MM cell line MOPC315.BM was resistant to direct MYXV binding and infection when tested in vitro. However, the delivery of MYXV with ex vivo loaded allogeneic BM leukocytes in the mice pre-seeded with MM disease, dramatically reduced residual MM in the BM and spleen and extended the survival of mice [29]. These results suggest that MYXV is safe for hematopoietic stem cells and can be used with current treatment modalities for MM, such as monoclonal antibodies, adoptive cell therapies and immune checkpoint inhibitors. 

## 7. Enhancing Oncolytic Activity of MYXV by Genetic Manipulation 

It was reported that the VACV-encoded F11L protein promotes virus release and rapid spread by inhibiting GTPase RhoA signaling and its effector mDia, which results in a disruption of the cortical actin layer [99,100]. Unlike VACV, MYXV produces foci on monolayers of cultured cells rather than plaques, that instead resemble a focus of transformed cells. This is likely because MYXV lacks a homolog of the *F11L* gene. Expression of *F11L* in MYXV resulted in the formation of large viral plaques, resulting in high titers in multistep growth conditions, all due to the modulation of cortical actin [101]. The recombinant F11L protein expressing MYXV replicated to higher levels in several human cancer cells and induced better tumor control and prolonged survival of mice. The virus was also able to spread to a second, untreated tumor-bearing site. In addition, pharmacological inhibition, or siRNA-mediated silencing of key regulators of cortical actin RhoA, RhoC, mDia1 or limk2, also enhanced the oncolytic activity of MYXV [102].

Due to its the ability to carry large insertions of foreign DNA, MYXV has also been exploited as gene therapy delivery system with the potential to target essential cancer genes. With this goal, a recombinant MYXV was generated with a CRISPR/Cas9-based gene editing capability. Using this recombinant virus, the treatment of preclinical ERMS tumor xenograft with an NRAS-targeting version of MYXV significantly reduced tumor growth and increased overall survival [31]. As described before, MYXV has been used to express immune-stimulating cytokine IL-15 and IL-15Rα-IL-15, which resulted in enhanced innate and adaptive antitumor immune responses [27,28]. MYXV can also be used to target the PD1/PDL1 pathway within the TME, by the direct expression of single chain antibodies or soluble scavenger proteins. MYXV construct, expressing a soluble ectodomain form of PD1 (sPD1) from virus-infected cells, induced and maintained antitumor cytotoxic T cell responses within the tumor bed, which was safer and more effective than combination therapy with anti-PD1 antibody plus wild-type MYXV [103]. This result opened up the possibility of direct MYXV-mediated expression of antibodies/inhibitors for immune checkpoint inhibitor anti-cytotoxic T lymphocyte antigen 4 (CTLA4) and recently identified co-inhibitory receptors, such as lymphocyte activation gene 3 (*LAG-3*) and T cell immunoglobulin and mucin receptor protein 3 (TIM3) [104]. Based on the reported studies, other immune-stimulating molecules that can be expressed using MYXV are cytokines, such as granulocyte-macrophage colony-stimulating factor (GM-CSF), IL-12, IL-2, IL-21, and activators of co-stimulatory receptors, such as 4-1BB, OX40, Glucocorticoid-induced TNFR-related protein (GITR) and B7-1 (CD80) [104,105]. All these molecules have proven antitumor activity, and using viruses’ tumor-localized expression of these immune-stimulating transgenes or targeted inhibition of pathways can further improve the therapeutic outcome with OVs.

## 8. Conclusions and Future Directions

**Figure 1:**
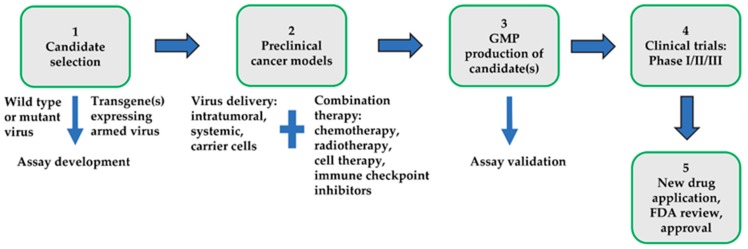
The clinical developmental path for the oncolytic virus. Multiple steps are involved in the selection, testing and development of an oncolytic virus candidate that can be successfully used in the clinic for cancer treatment.

MYXV is a promising candidate OV that has been successfully tested in many preclinical cancer models. In addition, the extreme safety profile of this virus outside of rabbits, the highly selective tropism for many diverse classes of cancer cells, and the restriction of virus replication in primary non-transformed human cells make MYXV an attractive OV platform. In order to render MYXV even safer for the one known susceptible host (European rabbits), targeted viral gene knockout mutant MYXV constructs are available, which retained the ability to infect and kill cancer cells but have lost the ability to cause any overt disease, even in rabbits [106,107]. Another advantage of MYXV is the ability to insert and express multiple therapeutic transgenes without compromising virus replication and production. This allowed the construction of multi-armed MYXV constructs that can express therapeutic transgenes directly in the tumor bed. In the future, the insertion of more therapeutic transgenes can further improve the extent of anti-tumor immune responses needed for the long-term clearance of tumors (Figure 1). At present, the main challenge with OV therapy in general is the systemic delivery of the virus and targeting the disseminated metastatic tumor sites. Initial studies have shown promising results for ex vivo virotherapy with MYXV by exploiting different types of either autologous or allogeneic carrier leukocytes, with the ability to deliver the virus to hard-to-reach metastatic sites. Establishing the optimal carrier cells for delivering MYXV to diverse metastatic sites will further support the clinical development of MYXV for later stage disseminated cancers.

## Figures and Tables

**Table 1:** Summary of preclinical cancer models tested with oncolytic MYXV.

Type of Cancers	Animal Model	Tumor Establishment	MYXV Constructs and Delivery	Combination Therapy	Outcome (Ref)
Small cell lung cancer (SCLC)	C57BL/6 (p53^lox/loxp^ p130^lox2722/lox2722^ Rb^loxP/loxP^)	Intratracheal injection of adenovirus expressing Cre-recombinase	vMyx-M135KO, intranasal instillation	Virus alone and with cisplatin	Prolonged survival of mice when combined with cisplatin [22]
	NSG	Subcutaneous xenograft of human primary SCLC cells	Wild type (WT), intratumoral	none	Virus replication and extensive tumor necrosis [22]
	C57BL/6	Subcutaneous xenograft of mouse SCLC cells	WT, intratumoral	none	Virus replication, extensive tumor necrosis and CD45^+^ immune cell infiltration [22]
Ovarian cancer (OC)	C57BL/6	Murine OC cells in intraperitoneal cavity	WT and vMyx-M062RKO, intraperitoneal cavity	Virus alone and with cisplatin	Prolonged survival of mice when combined with cisplatin [23]
Glioblastoma (GBM)	C57BL/6J	Intracranial injection of murine BTICs	WT and vMyx-M11KO, intratumoral	Virus alone and with temozolomide	Prolonged survival of mice when combined with temozolomide [24]
Gallbladder cancer (GBC)	CD-1 nude	Subcutaneous xenograft of human GBC cells	WT, intratumoral	Virus alone and with Rapamycin or hyaluronan	Combination treatment with hyaluronan reduced tumor burden and prolonged survival [25]
Melanoma	C57BL/6 RAG^-/-^	Intracranial injection of mouse B16.SIY melanoma cells	WT, intratumoral	Virus alone, with rapamycin and activated T cells	Prolonged survival of mice when combined all the treatments [26]
	C57BL/6	Subcutaneous injection of B16F10 cells	WT and vMyx-IL-15, intratumoral injection	none	Prolonged survival of mice treated with vMyx-IL15 [27]
	C57BL/6 and C57BL/6 RAG^-/-^	Subcutaneous injection of B16F10 cells	WT, vMyx-IL-15 and vMyx-IL-15Rα-IL-15 intratumoral injection	none	Prolonged survival of mice with increased infiltration of NK and CD8+ T cells to the tumor bed [28]
Multiple myeloma (MM)	BALB/c	Intravenous injection of mouse MOPC315.BM cells	WT, delivery of virus with total bone marrow	none	Prolonged survival of mice [29]
	BALB/c	Intravenous injection of mouse MOPC315 cells	WT, systemic delivery	none	Prolonged survival of mice [30]
Embryonal rhabdomyosarcoma (ERMS)	NSG	Subcutaneous xenograft of human ERMS cells	WT and NRAS targeting CRISPR-Cas9 engineered MYXV, intratumoral injection	none	Reduced tumor volume and prolonged mice survival [31]

## References

[1] Lawler S.E., Speranza M.C., Cho C.F., Chiocca E.A. (2017). Oncolytic Viruses in Cancer Treatment: A Review. JAMA Oncol..

[2] Bommareddy P.K., Shettigar M., Kaufman H.L. (2018). Integrating oncolytic viruses in combination cancer immunotherapy. Nat. Rev. Immunol..

[3] Martinez-Quintanilla J., Seah I., Chua M., Shah K. (2019). Oncolytic viruses: Overcoming translational challenges. J. Clin. Investig..

[4] Bell J., McFadden G. (2014). Viruses for tumor therapy. Cell Host Microbe.

[5] Pol J.G., Levesque S., Workenhe S.T., Gujar S., Le Boeuf F., Clements D.R., Fahrner J.E., Fend L., Bell J.C., Mossman K.L. (2018). Trial Watch: Oncolytic viro-immunotherapy of hematologic and solid tumors. Oncoimmunology.

[6] Martin N.T., Bell J.C. (2018). Oncolytic Virus Combination Therapy: Killing One Bird with Two Stones. Mol. Ther. J. Am. Soc. Gene Ther..

[7] Eissa I.R., Bustos-Villalobos I., Ichinose T., Matsumura S., Naoe Y., Miyajima N., Morimoto D., Mukoyama N., Zhiwen W., Tanaka M. (2018). The Current Status and Future Prospects of Oncolytic Viruses in Clinical Trials against Melanoma, Glioma, Pancreatic, and Breast Cancers. Cancers.

[8] Wei D., Xu J., Liu X.Y., Chen Z.N., Bian H. (2018). Fighting Cancer with Viruses: Oncolytic Virus Therapy in China. Human Gene Ther..

[9] Raman S.S., Hecht J.R., Chan E. (2019). Talimogene laherparepvec: Review of its mechanism of action and clinical efficacy and safety. Immunotherapy.

[10] Stanford M.M., Werden S.J., McFadden G. (2007). Myxoma virus in the European rabbit: Interactions between the virus and its susceptible host. Vet. Res..

[11] Kerr P.J., Liu J., Cattadori I., Ghedin E., Read A.F., Holmes E.C. (2015). Myxoma virus and the Leporipoxviruses: An evolutionary paradigm. Viruses.

[12] Cameron C., Hota-Mitchell S., Chen L., Barrett J., Cao J.X., Macaulay C., Willer D., Evans D., McFadden G. (1999). The complete DNA sequence of myxoma virus. Virology.

[13] Barrett J.W., Cao J.X., Hota-Mitchell S., McFadden G. (2001). Immunomodulatory proteins of myxoma virus. Semin. Immunol..

[14] Seet B.T., Johnston J.B., Brunetti C.R., Barrett J.W., Everett H., Cameron C., Sypula J., Nazarian S.H., Lucas A., McFadden G. (2003). Poxviruses and immune evasion. Annu. Rev. Immunol..

[15] McFadden G. (2005). Poxvirus tropism. Nat. Rev. Microbiol..

[16] Bertagnoli S., Marchandeau S. (2015). Myxomatosis. Rev. Sci. Tech. (Int. Off. Epizoot.).

[17] Kerr P.J. (2012). Myxomatosis in Australia and Europe: A model for emerging infectious diseases. Antivir. Res..

[18] Fenner F., Day M.F., Woodroofe G.M. (1956). Epidemiological consequences of the mechanical transmission of myxomatosis by mosquitoes. J. Hyg..

[19] Fenner F., Woodroofe G.M. (1953). The pathogenesis of infectious myxomatosis; the mechanism of infection and the immunological response in the European rabbit (Oryctolagus cuniculus). Br. J. Exp. Pathol..

[20] Fenner F. (1956). Evolutionary aspects of Myxomatosis in Australia. Mem. Inst. Oswaldo Cruz.

[21] Chan W.M., Rahman M.M., McFadden G. (2013). Oncolytic myxoma virus: The path to clinic. Vaccine.

[22] Kellish P., Shabashvili D., Rahman M.M., Nawab A., Guijarro M.V., Zhang M., Cao C., Moussatche N., Boyle T., Antonia S. (2019). Oncolytic virotherapy for small-cell lung cancer induces immune infiltration and prolongs survival. J. Clin. Investig..

[23] Nounamo B., Liem J., Cannon M., Liu J. (2017). Myxoma Virus Optimizes Cisplatin for the Treatment of Ovarian Cancer In Vitro and in a Syngeneic Murine Dissemination Model. Mol. Ther. Oncolytics.

[24] Pisklakova A., McKenzie B., Zemp F., Lun X., Kenchappa R.S., Etame A.B., Rahman M.M., Reilly K., Pilon-Thomas S., McFadden G. (2016). M011L-deficient oncolytic myxoma virus induces apoptosis in brain tumor-initiating cells and enhances survival in a novel immunocompetent mouse model of glioblastoma. Neuro-Oncol..

[25] Weng M., Gong W., Ma M., Chu B., Qin Y., Zhang M., Lun X., McFadden G., Forsyth P., Yang Y. (2014). Targeting gallbladder cancer: Oncolytic virotherapy with myxoma virus is enhanced by rapamycin in vitro and further improved by hyaluronan in vivo. Mol. Cancer.

[26] Thomas D.L., Doty R., Tosic V., Liu J., Kranz D.M., McFadden G., Macneill A.L., Roy E.J. (2011). Myxoma virus combined with rapamycin treatment enhances adoptive T cell therapy for murine melanoma brain tumors. Cancer Immunol. Immunother..

[27] Doty R.A., Liu J., McFadden G., Roy E.J., MacNeill A.L. (2013). Histological evaluation of intratumoral myxoma virus treatment in an immunocompetent mouse model of melanoma. Oncolytic Virotherapy.

[28] Tosic V., Thomas D.L., Kranz D.M., Liu J., McFadden G., Shisler J.L., MacNeill A.L., Roy E.J. (2014). Myxoma virus expressing a fusion protein of interleukin-15 (IL15) and IL15 receptor alpha has enhanced antitumor activity. PLoS ONE.

[29] Lilly C.L., Villa N.Y., Lemos de Matos A., Ali H.M., Dhillon J.S., Hofland T., Rahman M.M., Chan W., Bogen B., Cogle C. (2017). Ex Vivo Oncolytic Virotherapy with Myxoma Virus Arms Multiple Allogeneic Bone Marrow Transplant Leukocytes to Enhance Graft versus Tumor. Mol. Ther. Oncolytics.

[30] Bartee E., Bartee M.Y., Bogen B., Yu X.Z. (2016). Systemic therapy with oncolytic myxoma virus cures established residual multiple myeloma in mice. Mol. Ther. Oncolytics.

[31] Phelps M.P., Yang H., Patel S., Rahman M.M., McFadden G., Chen E. (2018). Oncolytic Virus-Mediated RAS Targeting in Rhabdomyosarcoma. Mol. Ther. Oncolytics.

[32] Schmidt F.I., Bleck C.K., Mercer J. (2012). Poxvirus host cell entry. Curr. Opin. Virol..

[33] Kim M., Madlambayan G.J., Rahman M.M., Smallwood S.E., Meacham A.M., Hosaka K., Scott E.W., Cogle C.R., McFadden G. (2009). Myxoma virus targets primary human leukemic stem and progenitor cells while sparing normal hematopoietic stem and progenitor cells. Leukemia.

[34] Byrd D., Amet T., Hu N., Lan J., Hu S., Yu Q. (2013). Primary human leukocyte subsets differentially express vaccinia virus receptors enriched in lipid rafts. J. Virol..

[35] Moss B. (2012). Poxvirus cell entry: How many proteins does it take?. Viruses.

[36] Moss B. (2016). Membrane fusion during poxvirus entry. Semin. Cell Dev. Biol..

[37] Mercer J., Helenius A. (2008). Vaccinia virus uses macropinocytosis and apoptotic mimicry to enter host cells. Science.

[38] Mercer J., Knebel S., Schmidt F.I., Crouse J., Burkard C., Helenius A. (2010). Vaccinia virus strains use distinct forms of macropinocytosis for host-cell entry. Proc. Natl. Acad. Sci. USA.

[39] Hsiao J.C., Chung C.S., Chang W. (1999). Vaccinia virus envelope D8L protein binds to cell surface chondroitin sulfate and mediates the adsorption of intracellular mature virions to cells. J. Virol..

[40] Chiu W.L., Lin C.L., Yang M.H., Tzou D.L., Chang W. (2007). Vaccinia virus 4c (A26L) protein on intracellular mature virus binds to the extracellular cellular matrix laminin. J. Virol..

[41] Chung C.S., Hsiao J.C., Chang Y.S., Chang W. (1998). A27L protein mediates vaccinia virus interaction with cell surface heparan sulfate. J. Virol..

[42] Lin C.L., Chung C.S., Heine H.G., Chang W. (2000). Vaccinia virus envelope H3L protein binds to cell surface heparan sulfate and is important for intracellular mature virion morphogenesis and virus infection in vitro and in vivo. J. Virol..

[43] Izmailyan R., Hsao J.C., Chung C.S., Chen C.H., Hsu P.W., Liao C.L., Chang W. (2012). Integrin beta1 mediates vaccinia virus entry through activation of PI3K/Akt signaling. J. Virol..

[44] Schroeder N., Chung C.S., Chen C.H., Liao C.L., Chang W. (2012). The lipid raft-associated protein CD98 is required for vaccinia virus endocytosis. J. Virol..

[45] Chan W.M., Bartee E.C., Moreb J.S., Dower K., Connor J.H., McFadden G. (2013). Myxoma and vaccinia viruses bind differentially to human leukocytes. J. Virol..

[46] Villa N.Y., Bartee E., Mohamed M.R., Rahman M.M., Barrett J.W., McFadden G. (2010). Myxoma and vaccinia viruses exploit different mechanisms to enter and infect human cancer cells. Virology.

[47] Villa N.Y., Wasserfall C.H., Meacham A.M., Wise E., Chan W., Wingard J.R., McFadden G., Cogle C.R. (2015). Myxoma virus suppresses proliferation of activated T lymphocytes yet permits oncolytic virus transfer to cancer cells. Blood.

[48] Wang G., Barrett J.W., Stanford M., Werden S.J., Johnston J.B., Gao X., Sun M., Cheng J.Q., McFadden G. (2006). Infection of human cancer cells with myxoma virus requires Akt activation via interaction with a viral ankyrin-repeat host range factor. Proc. Natl. Acad. Sci. USA.

[49] Werden S.J., McFadden G. (2010). Pharmacological manipulation of the akt signaling pathway regulates myxoma virus replication and tropism in human cancer cells. J. Virol..

[50] Werden S.J., McFadden G. (2008). The role of cell signaling in poxvirus tropism: The case of the M-T5 host range protein of myxoma virus. Biochim. Biophys. Acta.

[51] Liu J., Wennier S., Zhang L., McFadden G. (2011). M062 is a host range factor essential for myxoma virus pathogenesis and functions as an antagonist of host SAMD9 in human cells. J. Virol..

[52] Meng X., Zhang F., Yan B., Si C., Honda H., Nagamachi A., Sun L.Z., Xiang Y. (2018). A paralogous pair of mammalian host restriction factors form a critical host barrier against poxvirus infection. PLoS Pathog..

[53] Zhang F., Meng X., Townsend M.B., Satheshkumar P.S., Xiang Y. (2019). Identification of CP77 as the Third Orthopoxvirus SAMD9 and SAMD9L Inhibitor with Unique Specificity for a Rodent SAMD9L. J. Virol..

[54] Sivan G., Ormanoglu P., Buehler E.C., Martin S.E., Moss B. (2015). Identification of Restriction Factors by Human Genome-Wide RNA Interference Screening of Viral Host Range Mutants Exemplified by Discovery of SAMD9 and WDR6 as Inhibitors of the Vaccinia Virus K1L-C7L- Mutant. MBio.

[55] Meng X., Krumm B., Li Y., Deng J., Xiang Y. (2015). Structural basis for antagonizing a host restriction factor by C7 family of poxvirus host-range proteins. Proc. Natl. Acad. Sci. USA.

[56] Rahman M.M., Liu J., Chan W.M., Rothenburg S., McFadden G. (2013). Myxoma virus protein M029 is a dual function immunomodulator that inhibits PKR and also conscripts RHA/DHX9 to promote expanded host tropism and viral replication. PLoS Pathog..

[57] Rahman M.M., Bagdassarian E., Ali M.A.M., McFadden G. (2017). Identification of host DEAD-box RNA helicases that regulate cellular tropism of oncolytic Myxoma virus in human cancer cells. Sci. Rep..

[58] Kim M., Williamson C.T., Prudhomme J., Bebb D.G., Riabowol K., Lee P.W., Lees-Miller S.P., Mori Y., Rahman M.M., McFadden G. (2010). The viral tropism of two distinct oncolytic viruses, reovirus and myxoma virus, is modulated by cellular tumor suppressor gene status. Oncogene.

[59] Bartee E., McFadden G. (2009). Human cancer cells have specifically lost the ability to induce the synergistic state caused by tumor necrosis factor plus interferon-beta. Cytokine.

[60] Bartee E., Mohamed M.R., Lopez M.C., Baker H.V., McFadden G. (2009). The addition of tumor necrosis factor plus beta interferon induces a novel synergistic antiviral state against poxviruses in primary human fibroblasts. J. Virol..

[61] Veyer D.L., Carrara G., Maluquer de Motes C., Smith G.L. (2017). Vaccinia virus evasion of regulated cell death. Immunol. Lett..

[62] Davola M.E., Mossman K.L. (2019). Oncolytic viruses: How "lytic" must they be for therapeutic efficacy?. Oncoimmunology.

[63] Bartee M.Y., Dunlap K.M., Bartee E. (2016). Myxoma Virus Induces Ligand Independent Extrinsic Apoptosis in Human Myeloma Cells. Clin. Lymphoma Myeloma Leuk..

[64] Bartee E., Chan W.M., Moreb J.S., Cogle C.R., McFadden G. (2012). Selective purging of human multiple myeloma cells from autologous stem cell transplantation grafts using oncolytic myxoma virus. Biol. Blood Marrow Transplant..

[65] Everett H., Barry M., Lee S.F., Sun X., Graham K., Stone J., Bleackley R.C., McFadden G. (2000). M11L: A novel mitochondria-localized protein of myxoma virus that blocks apoptosis of infected leukocytes. J. Exp. Med..

[66] Kvansakul M., van Delft M.F., Lee E.F., Gulbis J.M., Fairlie W.D., Huang D.C., Colman P.M. (2007). A structural viral mimic of prosurvival Bcl-2: A pivotal role for sequestering proapoptotic Bax and Bak. Mol. Cell.

[67] Wang G., Barrett J.W., Nazarian S.H., Everett H., Gao X., Bleackley C., Colwill K., Moran M.F., McFadden G. (2004). Myxoma virus M11L prevents apoptosis through constitutive interaction with Bak. J. Virol..

[68] Douglas A.E., Corbett K.D., Berger J.M., McFadden G., Handel T.M. (2007). Structure of M11L: A myxoma virus structural homolog of the apoptosis inhibitor, Bcl-2. Protein Sci. A Publ. Protein Soc..

[69] Johnston J.B., Barrett J.W., Nazarian S.H., Goodwin M., Ricciuto D., Wang G., McFadden G. (2005). A poxvirus-encoded pyrin domain protein interacts with ASC-1 to inhibit host inflammatory and apoptotic responses to infection. Immunity.

[70] Macen J.L., Upton C., Nation N., McFadden G. (1993). SERP1, a serine proteinase inhibitor encoded by myxoma virus, is a secreted glycoprotein that interferes with inflammation. Virology.

[71] Turner P.C., Sancho M.C., Thoennes S.R., Caputo A., Bleackley R.C., Moyer R.W. (1999). Myxoma virus Serp2 is a weak inhibitor of granzyme B and interleukin-1beta-converting enzyme in vitro and unlike CrmA cannot block apoptosis in cowpox virus-infected cells. J. Virol..

[72] Urbasic A.S., Hynes S., Somrak A., Contakos S., Rahman M.M., Liu J., MacNeill A.L. (2012). Oncolysis of canine tumor cells by myxoma virus lacking the serp2 gene. Am. J. Vet. Res..

[73] Yang D., Denny S.K., Greenside P.G., Chaikovsky A.C., Brady J.J., Ouadah Y., Granja J.M., Jahchan N.S., Lim J.S., Kwok S. (2018). Intertumoral Heterogeneity in SCLC Is Influenced by the Cell Type of Origin. Cancer Discov..

[74] Sun A., Durocher-Allen L.D., Ellis P.M., Ung Y.C., Goffin J.R., Ramchandar K., Darling G. (2019). Initial management of small-cell lung cancer (limited- and extensive-stage) and the role of thoracic radiotherapy and first-line chemotherapy: A systematic review. Curr. Oncol..

[75] Armstrong S.A., Liu S.V. (2019). Immune Checkpoint Inhibitors in Small Cell Lung Cancer: A Partially Realized Potential. Adv. Ther..

[76] Dash A.S., Patel M.R. (2017). Viroimmunotherapy of Thoracic Cancers. Biomedicines.

[77] Schaffer B.E., Park K.S., Yiu G., Conklin J.F., Lin C., Burkhart D.L., Karnezis A.N., Sweet-Cordero E.A., Sage J. (2010). Loss of p130 accelerates tumor development in a mouse model for human small-cell lung carcinoma. Cancer Res..

[78] de Queiroz N., Xia T., Konno H., Barber G.N. (2019). Ovarian Cancer Cells Commonly Exhibit Defective STING Signaling Which Affects Sensitivity to Viral Oncolysis. Mol. Cancer Res..

[79] Hoare J., Campbell N., Carapuca E. (2018). Oncolytic virus immunotherapies in ovarian cancer: Moving beyond adenoviruses. Porto Biomed. J..

[80] Correa R.J., Komar M., Tong J.G., Sivapragasam M., Rahman M.M., McFadden G., Dimattia G.E., Shepherd T.G. (2012). Myxoma virus-mediated oncolysis of ascites-derived human ovarian cancer cells and spheroids is impacted by differential AKT activity. Gynecol. Oncol..

[81] Tong J.G., Valdes Y.R., Barrett J.W., Bell J.C., Stojdl D., McFadden G., McCart J.A., DiMattia G.E., Shepherd T.G. (2015). Evidence for differential viral oncolytic efficacy in an in vitro model of epithelial ovarian cancer metastasis. Mol. Ther. Oncolytics.

[82] Zhang H., Wang R., Yu Y., Liu J., Luo T., Fan F. (2019). Glioblastoma Treatment Modalities besides Surgery. J. Cancer.

[83] Martikainen M., Essand M. (2019). Virus-Based Immunotherapy of Glioblastoma. Cancers.

[84] Lun X., Yang W., Alain T., Shi Z.Q., Muzik H., Barrett J.W., McFadden G., Bell J., Hamilton M.G., Senger D.L. (2005). Myxoma virus is a novel oncolytic virus with significant antitumor activity against experimental human gliomas. Cancer Res..

[85] Lun X., Alain T., Zemp F.J., Zhou H., Rahman M.M., Hamilton M.G., McFadden G., Bell J., Senger D.L., Forsyth P.A. (2010). Myxoma virus virotherapy for glioma in immunocompetent animal models: Optimizing administration routes and synergy with rapamycin. Cancer Res..

[86] Zemp F.J., Lun X., McKenzie B.A., Zhou H., Maxwell L., Sun B., Kelly J.J., Stechishin O., Luchman A., Weiss S. (2013). Treating brain tumor-initiating cells using a combination of myxoma virus and rapamycin. Neuro-Oncology.

[87] McKenzie B.A., Zemp F.J., Pisklakova A., Narendran A., McFadden G., Lun X., Kenchappa R.S., Kurz E.U., Forsyth P.A. (2015). In vitro screen of a small molecule inhibitor drug library identifies multiple compounds that synergize with oncolytic myxoma virus against human brain tumor-initiating cells. Neuro-Oncology.

[88] Zemp F.J., McKenzie B.A., Lun X., Maxwell L., Reilly K.M., McFadden G., Yong V.W., Forsyth P.A. (2013). Resistance to oncolytic myxoma virus therapy in nf1(-/-)/trp53(-/-) syngeneic mouse glioma models is independent of anti-viral type-I interferon. PLoS ONE.

[89] Rawla P., Sunkara T., Thandra K.C., Barsouk A. (2019). Epidemiology of gallbladder cancer. Clin. Exp. Hepatol..

[90] Weng M., Zhang M., Qin Y., Gong W., Tang Z., Quan Z., Wu K. (2014). Targeting gallbladder carcinoma: Bone marrow-derived stem cells as therapeutic delivery vehicles of myxoma virus. Chin. Med. J..

[91] Antohe M., Nedelcu R.I., Nichita L., Popp C.G., Cioplea M., Brinzea A., Hodorogea A., Calinescu A., Balaban M., Ion D.A. (2019). Tumor infiltrating lymphocytes: The regulator of melanoma evolution. Oncol. Lett..

[92] Bayan C.Y., Lopez A.T., Gartrell R.D., Komatsubara K.M., Bogardus M., Rao N., Chen C., Hart T.D., Enzler T., Rizk E.M. (2018). The Role of Oncolytic Viruses in the Treatment of Melanoma. Curr. Oncol. Rep..

[93] Stanford M.M., Shaban M., Barrett J.W., Werden S.J., Gilbert P.A., Bondy-Denomy J., Mackenzie L., Graham K.C., Chambers A.F., McFadden G. (2008). Myxoma virus oncolysis of primary and metastatic B16F10 mouse tumors in vivo. Mol. Ther. J. Am. Soc. Gene Ther..

[94] Bonaventura P., Shekarian T., Alcazer V., Valladeau-Guilemond J., Valsesia-Wittmann S., Amigorena S., Caux C., Depil S. (2019). Cold Tumors: A Therapeutic Challenge for Immunotherapy. Front. Immunol..

[95] Bais S., Bartee E., Rahman M.M., McFadden G., Cogle C.R. (2012). Oncolytic virotherapy for hematological malignancies. Adv. Virol..

[96] Rahman M.M., Madlambayan G.J., Cogle C.R., McFadden G. (2010). Oncolytic viral purging of leukemic hematopoietic stem and progenitor cells with Myxoma virus. Cytokine Growth Factor Rev..

[97] Madlambayan G.J., Bartee E., Kim M., Rahman M.M., Meacham A., Scott E.W., McFadden G., Cogle C.R. (2012). Acute myeloid leukemia targeting by myxoma virus in vivo depends on cell binding but not permissiveness to infection in vitro. Leuk. Res..

[98] Russell S.J., Federspiel M.J., Peng K.W., Tong C., Dingli D., Morice W.G., Lowe V., O’Connor M.K., Kyle R.A., Leung N. (2014). Remission of disseminated cancer after systemic oncolytic virotherapy. Mayo Clin. Proc..

[99] Valderrama F., Cordeiro J.V., Schleich S., Frischknecht F., Way M. (2006). Vaccinia virus-induced cell motility requires F11L-mediated inhibition of RhoA signaling. Science.

[100] Arakawa Y., Cordeiro J.V., Way M. (2007). F11L-mediated inhibition of RhoA-mDia signaling stimulates microtubule dynamics during vaccinia virus infection. Cell Host Microbe.

[101] Irwin C.R., Evans D.H. (2012). Modulation of the myxoma virus plaque phenotype by vaccinia virus protein F11. J. Virol..

[102] Irwin C.R., Favis N.A., Agopsowicz K.C., Hitt M.M., Evans D.H. (2013). Myxoma virus oncolytic efficiency can be enhanced through chemical or genetic disruption of the actin cytoskeleton. PLoS ONE.

[103] Bartee M.Y., Dunlap K.M., Bartee E. (2017). Tumor-Localized Secretion of Soluble PD1 Enhances Oncolytic Virotherapy. Cancer Res..

[104] Harrington K., Freeman D.J., Kelly B., Harper J., Soria J.C. (2019). Optimizing oncolytic virotherapy in cancer treatment. Nat. Rev. Drug Discov..

[105] De Graaf J.F., de Vor L., Fouchier R.A.M., van den Hoogen B.G. (2018). Armed oncolytic viruses: A kick-start for anti-tumor immunity. Cytokine Growth Factor Rev..

[106] Barrett J.W., Alston L.R., Wang F., Stanford M.M., Gilbert P.A., Gao X., Jimenez J., Villeneuve D., Forsyth P., McFadden G. (2007). Identification of host range mutants of myxoma virus with altered oncolytic potential in human glioma cells. J. Neurovirol..

[107] Liu J., Wennier S., McFadden G. (2010). The immunoregulatory properties of oncolytic myxoma virus and their implications in therapeutics. Microbes Infect..

